# Tirzepatide‐Induced Functional Small Bowel Obstruction in a Surgically Naïve Patient: A Case Report

**DOI:** 10.1002/ccr3.73085

**Published:** 2026-07-02

**Authors:** Takuto Shimada, Yoshihiro Nose, Hiroki Tojima, Takahiro Maehata, Akitoshi Kato

**Affiliations:** ^1^ Department of Surgery Ogaki Municipal Hospital Gifu Japan

**Keywords:** case report, drug‐induced ileus, GLP‐1/GIP receptor agonist, small bowel obstruction, tirzepatide

## Abstract

Tirzepatide dose escalation may precipitate functional small bowel obstruction that mimics mechanical obstruction, even in surgically naïve patients. Careful medication review and objective tools such as the water‐soluble contrast challenge can support safe non‐operative management and help avoid unnecessary surgical intervention.

**Abbreviations**
GIP: glucose‐dependent insulinotropic polypeptide
GLP‐1: glucagon‐like peptide‐1
SBO: small bowel obstruction

## 1 Introduction

Tirzepatide is a dual glucose‐dependent insulinotropic polypeptide (GIP) and glucagon‐like peptide‐1 (GLP‐1) receptor agonist that is increasingly used in the management of type 2 diabetes and obesity. Adverse gastrointestinal effects, such as nausea, constipation, and delayed gastric emptying, are well recognized [1, 2]. Recently, a large pharmacovigilance study reported increased gastrointestinal adverse events, including bowel obstruction, with GLP‐1 receptor agonists [3]. Several case reports have also described mechanical or functional bowel obstruction associated with tirzepatide shortly after dose escalation [4, 5, 6].

However, the evidence guiding management decisions, including the role of water‐soluble contrast evaluation, remains limited. Herein, we describe a case of small bowel obstruction that occurred soon after tirzepatide dose escalation in which a structured contrast challenge interpreted using the Mori classification [7] allowed for safe nonoperative management.

## 2 Case History/Examination

A 57‐year‐old man with type 2 diabetes mellitus presented to the emergency department with acute abdominal pain. His medications included metformin, dapagliflozin, imeglimin, and telmisartan. The patient had no significant family history of gastrointestinal disease and no relevant psychosocial factors.

Tirzepatide therapy had been initiated at 2.5 mg once weekly. After 8 weeks of treatment, the dose was escalated to 5.0 mg according to the standard titration protocol. 9 days after this dose escalation, the patient developed sudden abdominal pain. The patient had a surgically naïve abdomen. His only surgical history was high inguinal orchiectomy for seminoma performed 15 years earlier via a scrotal approach, which did not involve entry into the abdominal cavity.

Upon arrival, the patient was afebrile and hemodynamically stable. Abdominal examination revealed mild distension and localized right‐sided tenderness. Laboratory investigations revealed no leukocytosis, elevated lactate levels, or metabolic acidosis.

**FIGURE 1:**
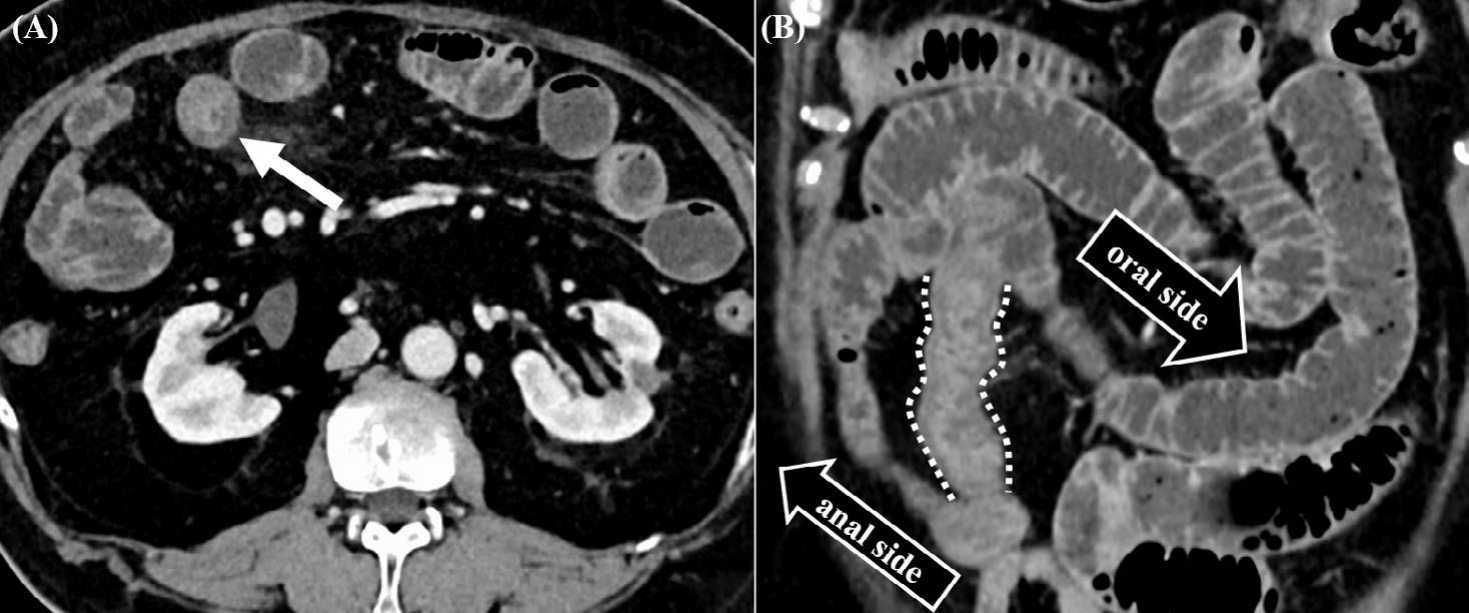
Contrast‐enhanced CT findings on admission. (A) Axial CT image demonstrating a transition point (arrow) in the small bowel. (B) The affected bowel segment (outlined with a dashed line) shows segmental mural thickening and luminal narrowing. The distal (anal side) bowel is collapsed, whereas the proximal (oral side) bowel is dilated with a small bowel feces sign. These findings mimic mechanical small bowel obstruction.

Contrast‐enhanced CT demonstrated proximal small bowel dilatation with a distinct transition point in the right abdomen (Figure 1). Differential diagnoses included mechanical obstruction due to internal hernia, inflammatory enteritis, and medication‐induced intestinal hypomotility. No signs of ischemia, closed‐loop obstruction, pneumatosis, or ascites were observed. The clinical findings were consistent with drug‐induced ileus.

## 3 Treatment and Follow‐Up

**FIGURE 2:**
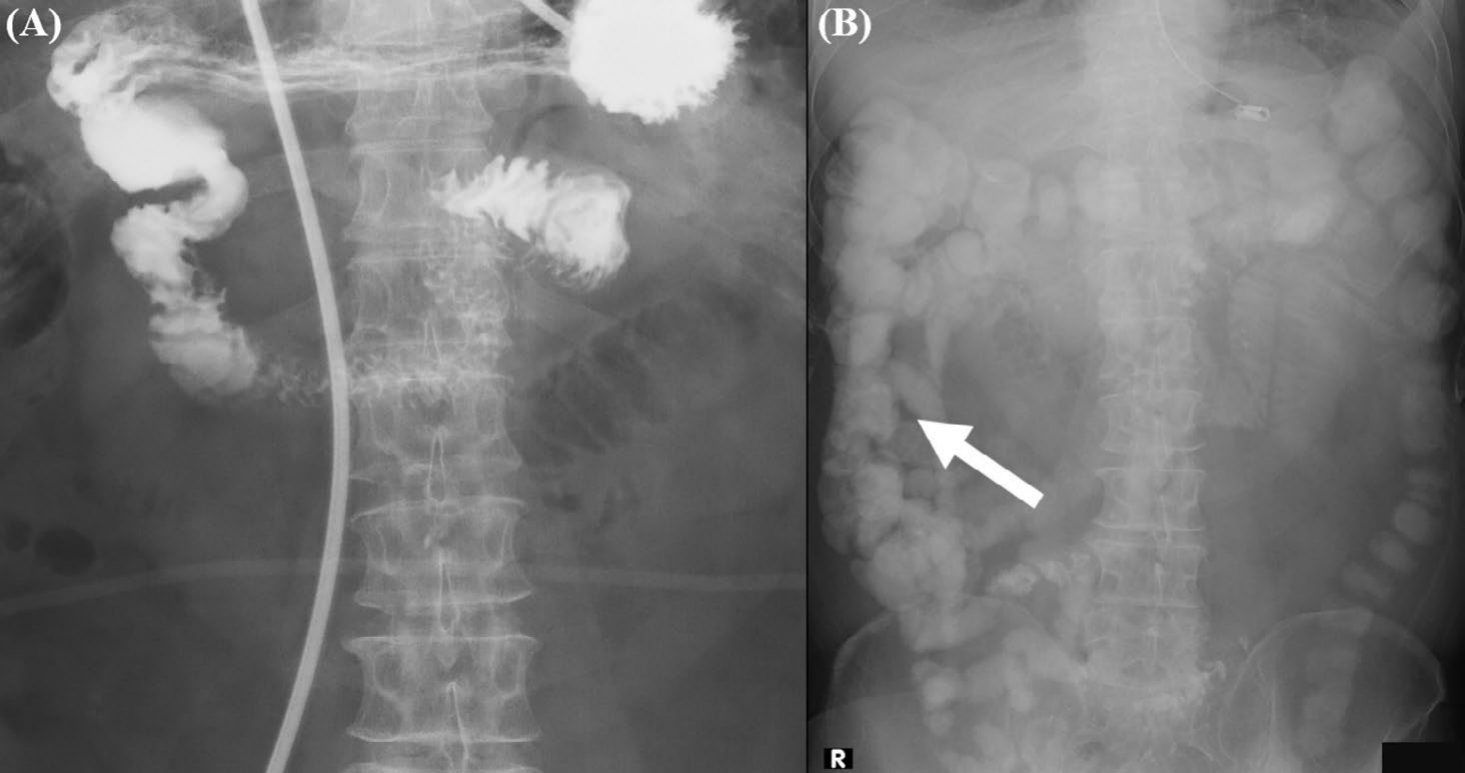
Water‐soluble contrast challenge using Gastrografin. (A) Abdominal radiograph immediately after administration via nasogastric tube. (B) Radiograph obtained 3 h later demonstrates contrast medium reaching the ascending colon (arrow), consistent with type 3b according to the Mori classification, indicating a high likelihood of successful conservative management.

A nasogastric tube was placed for decompression. Subsequently, a water‐soluble contrast challenge using gastrografin was performed. At 3 h, the contrast medium was observed in the colon (Figure 2). According to the Mori classification [7], this finding represents type 3b, which predicts a high likelihood of successful conservative management.

Therefore, the patient was treated with bowel rest, nasogastric decompression, and intravenous fluid administration. The tirzepatide treatment was discontinued. Over the next 48 h, the symptoms improved, nasogastric output decreased, and bowel movements resumed. The patient tolerated oral intake and was discharged on day 7. Tirzepatide therapy remained discontinued. No recurrence of obstructive symptoms was observed during 6 months of follow‐up.

## 4 Discussion

This case illustrates a clinical scenario in which drug‐induced intestinal hypomotility closely mimicked mechanical small bowel obstruction. Although the causal inference cannot be definitive, temporal proximity suggests that tirzepatide‐induced gastrointestinal hypomotility contributed to the obstruction. GLP‐1 receptor stimulation slows gastric emptying and suppresses migrating motor complexes, reducing small bowel transit [1, 2]. These effects were most pronounced during the initiation and dose escalation, which paralleled the patient's presentation.

Recent reports have described small or large bowel obstruction shortly after tirzepatide dose changes [4, 5, 6]. The temporal relationship between dose escalation and symptom onset is notable. Tirzepatide has a half‐life of approximately 5 days, and plasma concentrations gradually increase following dose escalation before reaching a new steady state over several weeks. During this period of rising drug exposure, the inhibitory effects on gastrointestinal motility may become more pronounced.

The radiologic findings in this case were particularly challenging. Computed tomography demonstrated an apparent transition point, a feature usually associated with mechanical obstruction. Such findings often prompt early surgical exploration, particularly in patients without prior abdominal surgery. However, this case demonstrates that drug‐induced intestinal hypomotility may produce imaging findings that closely resemble mechanical SBO.

Segmental mural thickening was also observed. Although bowel wall thickening can suggest inflammatory or ischemic pathology, transient thickening may occur in the setting of intestinal stasis and venous congestion associated with functional obstruction.

The water‐soluble contrast challenge played a key role in clinical decision‐making. This method has been widely validated as a diagnostic and therapeutic tool in small bowel obstruction [7, 8]. Rapid passage of contrast into the colon strongly indicated preserved intestinal continuity and supported continuation of conservative management.

The clinical course further supports a medication‐related mechanism. Symptoms occurred shortly after dose escalation and resolved following discontinuation of tirzepatide, with no recurrence during 6 months of follow‐up. These findings suggest that drug‐induced hypomotility likely contributed to the development of obstruction in this case.

From a practical perspective, this case highlights an important diagnostic pitfall. Drug‐induced intestinal hypomotility during incretin therapy may mimic mechanical SBO even on cross‐sectional imaging. Awareness of this possibility and careful medication review may help clinicians avoid unnecessary surgical intervention.

## 5 Conclusions

Tirzepatide dose escalation may precipitate functional small bowel obstruction that mimics mechanical obstruction even in surgically naïve patients. Recognition of this potential drug‐related complication and use of objective diagnostic tools such as the water‐soluble contrast challenge can support safe non‐operative management and help prevent unnecessary surgical exploration.

## Author Contributions

**Takuto Shimada:** conceptualization, investigation, writing – original draft, writing – review and editing. **Takahiro Maehata:** methodology. **Yoshihiro Nose:** supervision. **Akitoshi Kato:** conceptualization, writing – original draft. **Hiroki Tojima:** data curation.

## Funding

The authors have nothing to report.

## Ethics Statement

Ethics approval was not required for this case report in accordance with the local guidelines.

## Consent

Written informed consent was obtained from the patient for the publication of this case report and the accompanying images.

## Conflicts of Interest

The authors declare no conflicts of interest.

## Data Availability

The data that support the findings of this study are available on request from the corresponding author. The data are not publicly available due to privacy or ethical restrictions.
